# YOLOv7-CSAW for maritime target detection

**DOI:** 10.3389/fnbot.2023.1210470

**Published:** 2023-07-03

**Authors:** Qiang Zhu, Ke Ma, Zhong Wang, Peibei Shi

**Affiliations:** School of Computer Science and Technology, Hefei Normal University, Hefei, China

**Keywords:** maritime target detection, YOLOv7, SimAM, ASFF, WIoU

## Abstract

**Introduction:**

The issue of low detection rates and high false negative rates in maritime search and rescue operations has been a critical problem in current target detection algorithms. This is mainly due to the complex maritime environment and the small size of most targets. These challenges affect the algorithms' robustness and generalization.

**Methods:**

We proposed YOLOv7-CSAW, an improved maritime search and rescue target detection algorithm based on YOLOv7. We used the K-means++ algorithm for the optimal size determination of prior anchor boxes, ensuring an accurate match with actual objects. The C2f module was incorporated for a lightweight model capable of obtaining richer gradient flow information. The model's perception of small target features was increased with the non-parameter simple attention module (SimAM). We further upgraded the feature fusion network to an adaptive feature fusion network (ASFF) to address the lack of high-level semantic features in small targets. Lastly, we implemented the wise intersection over union (WIoU) loss function to tackle large positioning errors and missed detections.

**Results:**

Our algorithm was extensively tested on a maritime search and rescue dataset with YOLOv7 as the baseline model. We observed a significant improvement in the detection performance compared to traditional deep learning algorithms, with a mean average precision (mAP) improvement of 10.73% over the baseline model.

**Discussion:**

YOLOv7-CSAW significantly enhances the accuracy and robustness of small target detection in complex scenes. This algorithm effectively addresses the common issues experienced in maritime search and rescue operations, specifically improving the detection rates and reducing false negatives, proving to be a superior alternative to current target detection algorithms.

## 1. Introduction

According to statistics from the Chinese Ministry of Transport, in 2021, the China Maritime Search and Rescue Center organized a total of 1,824 search and rescue operations, involving ~12,258 people in distress, of which about 11,761 were rescued, and 1,214 vessels were saved.^1^ Rapid identification and location of maritime targets plays a crucial role in ensuring the safety of personnel and preventing property loss. Maritime target detection is widely used in search and rescue operations, such as shipwrecks, maritime disasters, and missing persons (Cho et al., 2021). Multiple sensors are used to collect ocean data, including optical and infrared cameras, drones (Wu et al., 2019), radars (Harzheim et al., 2021), tracking and navigation systems, etc. Additionally, satellite image analysis and aerial remote sensing technologies can be integrated to improve the accuracy of maritime search and rescue target detection.

With the rapid development of artificial intelligence and 5G communication technology, researchers have started using AI and machine learning techniques to carry out maritime rescue missions. Currently, common maritime search and rescue target detection algorithms include Faster Region-based Convolutional Neural Networks (Faster RCNN), You Only Look Once (YOLO), and Single Shot MultiBox Detector (SDD) (Yabin et al., 2020; Sambolek and Ivasic-Kos, 2021), which mainly use convolutional neural network algorithms to extract features from images and recognize potential search and rescue targets. End-to-end learning frameworks can also be used to achieve faster and more accurate target detection. These algorithms learn and train on a large amount of maritime image data to achieve efficient and accurate target detection.

YOLO is designed for real-time object detection, making it much faster than other object detection algorithms like Faster R-CNN and R-CNN Pascal VOC. The YOLO algorithm has been demonstrated to generalize well across various object categories and datasets. This ability to perform well on different types of objects is advantageous in rescue operations, where the targets of interest may include people, boats, and other objects in the marine environment. However, challenges still persist in sea rescue target detection due to factors such as the impact of marine environments on images, which can include elements like strong lighting, large amounts of water, waves, and refraction phenomena. Additionally, it is necessary to consider the appearance and features of targets, such as color, shape, and size, especially the problem of many small targets. In practical applications, there are still issues of missed detections and false alarms. To address these problems, this study improved the YOLOv7 algorithm (Wang et al., 2023), which has high speed and accuracy, for sea rescue target detection, with the following main contributions:

1. Utilizing the K-means++ algorithm to determine the optimal prior anchor box sizes, ensuring precise matching between anchor boxes and actual objects;
2. Enhancing the C3 module to C2f module, maintaining a lightweight design while obtaining more abundant gradient flow information;
3. Incorporating the parameter-free SimAM, bolstering the model's ability to perceive small target features;
4. Upgrading the feature fusion network to the ASFF, compensating for the missing high-level semantic features of small targets;
5. Employing the WIoU loss function to effectively address the issues of large positioning errors and missed detections, thereby enhancing the model's generalization ability.

The remaining content is arranged as follows: Section 2 introduces the related work of sea rescue target detection, Section 3 focuses on the improved YOLOv7 algorithm framework and implementation details, Section 4 verifies the performance of the proposed method through experimental testing, and finally provide a summary and outlook.

## 2. Related Works

Currently, numerous research achievements have been made in the field of maritime rescue. Yang et al. (2020) utilized unmanned aerial vehicles and unmanned surface vehicles to form a cognitive computing network for collaborative rescue and utilized reinforcement learning to plan and search for paths. Jin et al. (2021) modeled maritime rescue tasks using geographic information systems, then used K-means algorithm to obtain the location of search and rescue points, and finally input the obtained task point data into an optimization algorithm module. Ferrari and Chen (2020) treated the problem of planning a temporary fleet to perform search tasks in the open sea as a resource allocation problem and proposed a change to the objective function of the binary integer programming model to respond to different aspects of air search and rescue operations. Liu et al. (2021) constructed a new maritime target dataset (MRSP-13) and proposed a cross-layer, multi-task CNN model for maritime target detection, which is capable of concurrently addressing ship target detection, classification, and segmentation tasks. Gasienica-Jozkowy et al. (2021) publicly released a maritime rescue dataset and proposed an object detection method based on deep convolutional neural networks, which achieved an average precision of 82% on the dataset. Ai et al. (2021) established a maritime SAR environment model using field data of the marine environment and electronic charts, and proposed a reinforcement learning-based autonomous coverage path planning model for SAR missions, planning the shortest search path and prioritizing high probability areas. Zhou et al. (2020) proposed a comprehensive framework for evaluating maritime search and rescue capabilities, first estimating rescue response time through geographic information systems, then calculating search and rescue service demand based on relevant data, and finally determining evaluation standards to quantitatively evaluate maritime search and rescue capabilities. Xian et al. (2022) proposed an opportunistic routing protocol for low latency and energy-efficient wireless sensor networks for maritime search and rescue. Lu et al. (2020) propose a detection algorithm (SRGM) for infrared small dim targets in different maritime backgrounds. The algorithm uses an efficient maritime background filter to extract the image background accurately and eliminate it by comparing it to the original image.

Target detection in maritime search and rescue often involves small targets that occupy a small proportion of the entire image, making it difficult to extract their position and feature information. Therefore, maritime search and rescue target detection is also a small target detection problem. Deep learning-based small target detection algorithms mainly include improvements to backbone networks, adjustments to pyramid structures, and anchor box designs. Regarding backbone networks, the introduction of ResNet broke the limit on the number of neural network layers, promoting the development of deeper models, and several improved models such as ResNeXt (Xie et al., 2017), ResNeSt (Zhang H. et al., 2022), DarkNet (Bochkovskiy et al., 2020), and NFNet (Brock et al., 2021) emerged. Qiao et al. (2021) replaced the ResNet50 backbone network with ResNeXt-101 and achieved a 1.5% improvement in small target detection on the MSCOCO dataset. YOLOv4 introduced CSPNet (Wang et al., 2020) and designed CSP DarkNet-53 as the backbone network, reducing the number of backbone network parameters by 20%. MobileNet series (Howard et al., 2019) and ShuffleNet series (Zhang et al., 2018), representing lightweight backbone networks, optimize information exchange and parameter reduction. Liu et al. (2020) addressed the problem of small targets being easily lost in deep convolutional networks by designing an image pyramid network to enhance small target detection. To achieve repeated fusion of multi-scale information, Jiang et al. (2022) proposed a giraffe-like network called GiraffeDet, which includes two feature connection methods and achieves a 2.8% performance improvement in small target detection on the MSCOCO dataset. The current methods still struggle to achieve accurate detection for small targets consistently.

In addition to the backbone network, attention mechanisms can help neural networks focus on task-relevant details amidst a large amount of information. The SE attention mechanism proposed by Hu et al. (2018) focuses on more critical feature information by exploring the interdependence relationship between feature channels. Woo et al. (2018) designed the CBAM attention mechanism, which consists of a channel attention module and a spatial attention module, to enhance the network's feature extraction and strengthening ability. Wang et al. (2020) designed an efficient channel attention module called ECA, which enables direct local cross-channel interaction and significantly reduces the complexity associated with dimensionality reduction in channel learning. Yang et al. (2021) proposed the parameter-free attention module SimAM, which constructs an energy function to mine neuron importance based on neuroscience theory and derived an analytical solution to accelerate computation. Pan et al. (2022) proposed the ACmix attention module, which combines the advantages of Self-Attention and Convolution, and has a smaller computational overhead compared to models corresponding to Convolution or Self-Attention. Although attention mechanisms like SE, CBAM, ECA, SimAM, and ACmix have been developed to help neural networks focus on task-relevant information, further improvements are needed to optimize the feature extraction process without significantly increasing computational overhead.

While some studies have proposed frameworks for evaluating maritime search and rescue capabilities, more research is needed to develop robust and generalizable evaluation metrics that can assess the effectiveness of various algorithms and techniques in diverse maritime search and rescue scenarios. The YOLOv7 algorithm that our study utilized offers several advantages for maritime target detection, making it a suitable choice for addressing the challenges in this domain. YOLOv7 is designed to perform object detection at a high speed, making it capable of real-time detection in maritime scenarios. This is particularly beneficial for time-sensitive search and rescue operations. YOLOv7 incorporates various improvements over previous YOLO versions, such as enhanced backbone networks and better feature extraction mechanisms. These improvements contribute to higher accuracy in detecting maritime targets, including small targets that are difficult to detect. YOLOv7 utilizes a multi-scale detection approach, allowing it to effectively detect objects of different sizes in the same image. This is crucial for maritime target detection, as objects can appear at various scales due to factors like distance and perspective. YOLOv7 is designed for high performance with lower computational needs compared to other advanced object detection algorithms. This makes it ideal for use in resource-limited environments like unmanned aerial vehicles (UAVs) or unmanned surface vehicles (USVs) in maritime search and rescue operations. YOLOv7 can be easily fine-tuned and adapted to different maritime target detection scenarios using domain-specific training data. This adaptability enables the algorithm to consistently perform well across varying situations and environments in maritime search and rescue missions.

## 3. Methodology

### 3.1. Overview of YOLOv7

As a one-stage object detection algorithm, YOLOv7 outperforms most known object detectors within a range of 5FPS-160FPS in terms of both detection speed and accuracy. The YOLOv7 network model is composed of four parts: Input, Backbone, Neck, and Head. In the Input section, the image undergoes a series of pre-processing stages such as data augmentation before being fed into the Backbone network for feature extraction. Next, the extracted features are amalgamated by the Neck module to generate features of different sizes. These fused features are finally fed into the detection Head, which outputs the prediction results.

The backbone of YOLOv7 comprises several modules, including the CBS convolution layer, E-ELAN module, MPConv module, and SPPCSPC module. The E-ELAN module is a highly efficient layer aggregation network that enhances the learning ability of the network without disrupting the original gradient path. It also guides the calculation of different feature groups to learn more diverse features. The MPConv convolution layer adds a MaxPool layer to the CBS layer, forming upper and lower branches. These branches are then fused using the Concat operation to enhance the network's feature extraction ability. The SPPCSPC module introduces parallel MaxPool operations in a series of convolutions to prevent image distortion caused by image processing operations and to address the problem of extracting repeated features in convolutional neural networks.

The Neck module employs the traditional PAFPN structure and introduces a bottom-up path to facilitate the transfer of low-level information to higher levels, thus enabling efficient fusion of features at different levels. In the Head module, the image channel number of the PAFPN output features is adjusted using the REPConv structure, and predictions are made via convolution.

### 3.2. Improvement measures

#### 3.2.1. Anchor boxes

YOLOv7 utilizes K-means clustering to create anchor boxes, referencing the boundaries of the training set. Additionally, the FPN network establishes three distinct anchor boxes on three differing sizes of feature maps. However, the K-means algorithm has its inherent limitations, as it is prone to the influence of initial setting values and outliers, which can lead to unstable clustering results. Therefore, in this study, we have opted for the K-means++ clustering algorithm. By repeatedly clustering the annotated object boundaries in our dataset, we can produce prior boxes in a variety of numbers and sizes. This process enhances the degree of match between the prior boxes and the actual object boxes, which in turn improves the accuracy of detection.

#### 3.2.2. C2f module

**Figure 1:**
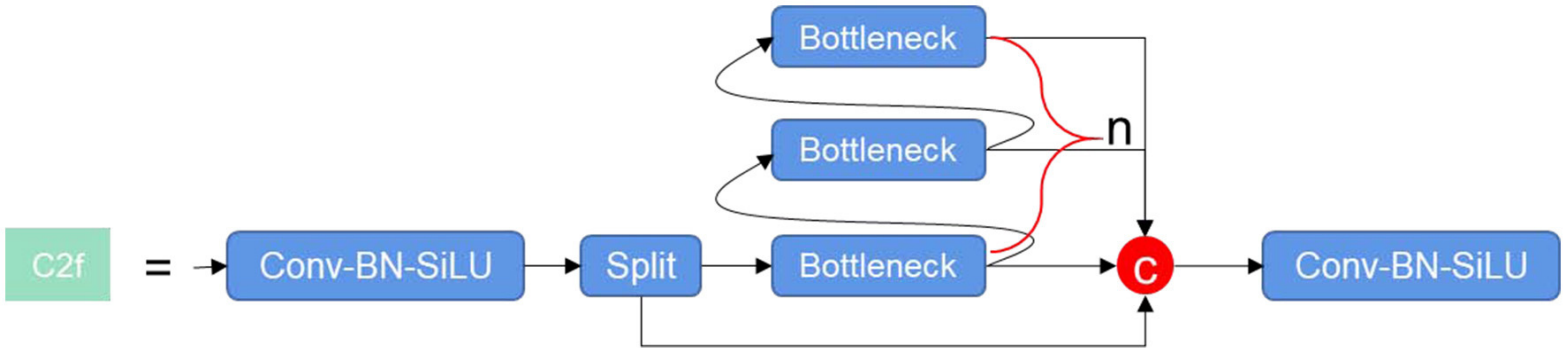
C2f module.

The C2f module,^2^ as depicted in Figure 1, has been architecturally designed, drawing inspiration from the C3 module and ELAN principles. This structure enables the enhanced network to glean more abundant gradient flow information, all the while maintaining a lightweight design. At the heart of this model, the Conv-BN-SiLU module carries out operations such as convolution, normalization, and activation on the input feature maps. The activation function is performed by SiLU. On the other hand, the Bottleneck module acts as a residual module, where the quantity of stacked layers is governed by the parameter “n”. This parameter changes according to the scale of different models.

The key responsibility of the Bottleneck module is to initially diminish the number of channels before proceeding to augment them. The usage of residual connections is managed by the shortcut parameter, with feature fusion being accomplished via the “add” operation. This approach ensures the number of features post-fusion remains consistent. By default, the C2f module does not utilize shortcut connections. While these connections are utilized in the Backbone, they are not incorporated in the Head.

#### 3.2.3. ASFF

**Figure 2:**
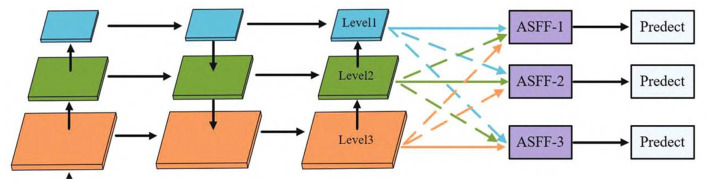
ASFF module.

ASFF (Liu et al., 2019) is a novel and effective feature fusion algorithm that enables the network to adaptively learn the weights of each position on each feature layer, so that important information dominates the feature fusion process. As shown in Figure 2, for each feature layer to be fused, other feature layers are first transformed to the same resolution, and then the optimal weights for fusion are learned through training. The principle involves several steps:

1. Feature Layer Alignment: Initially, ASFF equalizes the resolution of all feature layers that require fusion. This is accomplished either through interpolation or convolution operations, aiming to ensure that information from different feature layers can be compared and merged on the same spatial scale. This step is critical in small target detection, as small targets may exhibit different characteristics on feature maps of various scales. By aligning all feature layers to the same resolution, ASFF effectively captures and merges small target information across different scales.
2. Adaptive Weight Learning: After aligning the feature layers, ASFF learns the optimal fusion weights through training. This process is differentiable, meaning it can be adjusted via optimization algorithms like backpropagation and gradient descent. This feature proves very useful in small target detection, as the information of small targets may be more pronounced on certain feature layers. By adaptively learning the fusion weights, ASFF can accentuate these feature layers containing more small target information, allowing these details to dominate the feature fusion process.
3. Feature Fusion: Finally, based on the learned weights, ASFF fuses the features of each layer. Features with higher weights will dominate the fused feature representation. This implies that in small target detection, feature layers that are more capable of capturing small target information will be assigned greater weights, thus dominating the final feature representation.

In summary, ASFF can effectively enhance the detection of small maritime targets by adaptively adjusting the weights of each feature layer. This makes ASFF adaptable to various detection tasks, including the detection of small maritime targets, irrespective of the backbone network employed.

#### 3.2.4. SimAM

**Figure 3:**
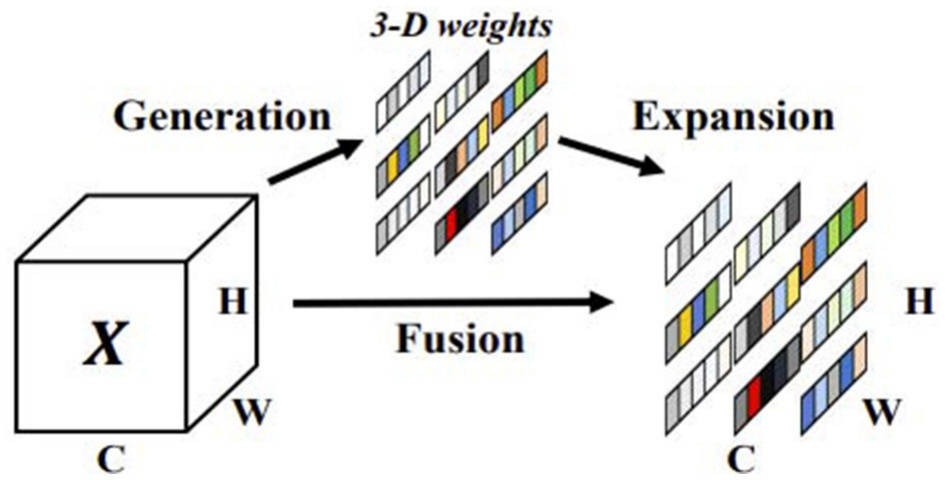
SimAM attention module.

SimAM is a parameter-free attention module that is based on neuroscience theory to identify important neurons. It constructs an energy function, as shown in the Figure 3, to better implement attention. To achieve attention, the SimAM module needs to evaluate the importance of each neuron. In neuroscience, neurons with rich information typically exhibit discharge patterns that are different from those of surrounding neurons (Webb et al., 2005; Hariharan et al., 2012). Furthermore, activated neurons often inhibit surrounding neurons, that is, spatial inhibition. In other words, neurons with spatial inhibition effects should be given higher importance. The simplest way to find important neurons is to measure the linear separability between neurons. Therefore, the following energy function is defined:


(1) $$\begin{array}{l} e_{t}\left(\omega_{t},b_{t},y,x_{i}\right)={\left(y_{t}-\hat{t}\right)}^{2}+\frac{1}{M-1}\sum_{i=1}^{M-1}{\left(y_{o}-{\hat{x}}_{i}\right)}^{2} \end{array}$$

where $\hat{t}=\omega_{t}t+b_{t}$ and ${\hat{x}}_{i}=\omega_{t}x_{i}+b_{t}$ are linear transforms of *t* and *x*_*i*_, *t* and *x*_*i*_ are the target neuron and other neurons in a single channel of the input feature. ω_*t*_ and *b*_*t*_ are weight and bias the transform. The Eqn (1) attains the minimal value when the $\hat{t}$ equals to *y*_*t*_, and all other ${\hat{x}}_{i}$ are *y*_*o*_, where *y*_*t*_ and *y*_*o*_ are two different values.

Minimizing the above formula is equivalent to training the linear separability between neuron t and other neurons within the same channel. For simplicity, binary labels are used and a regularization term is added. The final energy function is defined as follows:


(2) $$\begin{array}{l} e_{t}\left(\omega_{t},b_{t},y,x_{i}\right)=\frac{1}{M-1}\sum_{i=1}^{M-1}{\left(-1-\left(\omega_{t}x_{i}+b_{t}\right)\right)}^{2}\\ +\;{\left(1-\left(\omega_{t}t+b_{t}\right)\right)}^{2}+\lambda \omega_{t}^{2} \end{array}$$

In theory, there are *M* = *H*×*W* energy functions for each channel. The above formula has the following closed-form solution:


$$\begin{array}{l} \omega_{t}=-\frac{2\left(t-\mu_{t}\right)}{{\left(t-\mu_{t}\right)}^{2}+2\sigma_{t}^{2}+2\lambda } \end{array}$$

$$\begin{array}{l} b_{t}=-\frac{1}{2}\left(t+\mu t\right)\omega_{t} \end{array}$$

where $\mu_{t}=\frac{1}{M-1}\overset{M-1}{\underset{i=1}{\sum }}x_{i}\;$and $\sigma_{t}^{2}=\frac{1}{M-1}\overset{M-1}{\underset{i=1}{\sum }}{\left(x_{i}-\mu_{t}\right)}^{2}$ are mean and variance calculated over all neurons except *t* in that channel.

Since all neurons in each channel follow the same distribution, the mean and variance of the input feature can be computed first in the *H* and *W* dimensions to avoid redundant calculations:


(3) $$\begin{array}{l} e_{t}^{*}=\frac{4\left({\hat{\sigma }}^{2}+\lambda \right)}{{\left(t-\hat{\mu }\right)}^{2}+2{\hat{\sigma }}^{2}+2\lambda } \end{array}$$

where $\hat{\mu }=\frac{1}{M}\overset{M}{\underset{i=1}{\sum }}x_{i}\;$and ${\hat{\sigma }}^{2}=\frac{1}{M}\overset{M}{\underset{i=1}{\sum }}{\left(x_{i}-\;\hat{\mu }\right)}^{2}$

Equation (3) reveals an observation: the lower the energy of a neuron *t*, the greater its dissimilarity with surrounding neurons, and therefore, the more significant its contribution to visual processing. Thus, the importance of each neuron can be evaluated by $e_{t}^{*}$. Our approach involves operating on each neuron individually and integrating this linear separability into an end-to-end framework.

According to the definition of the attention mechanism, we need to enhance the features. The whole process can be expressed as:


(4) $$\begin{array}{l} \tilde{X}=sigmoid\left(\frac{1}{E}\right)⊙\;X \end{array}$$

where *E* groups all across channel and spatial dimensions. *sigmoid* is added to restrict too large value in *E*. It will not influence the relative importance of each neuron because *sigmoid* is a monofonic function.

In fact, aside from the calculation of channel averages and variances, all computations within the SimAM module are element-wise operations, which can be readily implemented using current machine learning libraries with just a few lines of code to accomplish the functionality of Equation (4). Thus, the SimAM module, rooted in fundamental neuroscience theories, distinguishes itself from other attention mechanisms. Moreover, the module can be efficiently integrated into existing network architectures without significantly increasing their complexity or computational requirements.

#### 3.2.5. WIoU

In traditional IoU computation, the IoU score for a predicted bounding box and a ground truth bounding box is calculated as the ratio of their intersecting area to their total area. However, this conventional method can sometimes lead to suboptimal results. For instance, smaller objects, given their fewer pixel counts, carry less weight in the IoU calculation, potentially causing the model to overlook these smaller entities due to bias. Wise IoU (Tong et al., 2023) addresses this issue by modifying the IoU loss function during the model training process. It introduces a dynamic adjustment factor that modifies the IoU value, taking into account factors such as the size of the object, occlusion, and the complexity of the background. This dynamic adjustment allows for more precise object detection, particularly for challenging cases like small objects or those in complex environments.

Assuming the anchor box is $\overset{⇀}{B}=\left[x\;y\;w\;h\right]$, and the ground truth box is ${\overset{⇀}{B}}_{gt}=\left[x_{gt}\;y_{gt}\;w_{gt}\;h_{gt}\right]$. IoU is used to measure the degree of overlap between the predicted frame and the real frame in the target detection task, defined as $L_{IoU}=1-IoU$. WIoU has three versions: WIoU v1 constructs a boundary box loss based on attention, while WIoU v2 and WIoU v3 attach a focusing mechanism by constructing gradient gain and using algorithmic methods on this basis.

##### 3.2.5.1. WIoU v1

Inevitably, training datasets contain low-quality examples. Geometric metrics such as distance and aspect ratio can intensify the penalty on these low-quality samples, ultimately undermining the model's generalization performance. An effective loss function should mitigate the penalty of geometric measures when the anchor box and the target box align well. However, excessive intervention during training can enhance the model's generalization capability. Building on this, distance attention is constructed based on the distance metric, resulting in a two-layer attention mechanism. This approach allows the model to more accurately attend to important features, thereby improving its ability to generalize from the training data to unseen examples.


(5) $${ℒ}_{WIoU\;v1}={ℛ}_{WIoU\;}{ℒ}_{IoU}=exp(\frac{{\left(x-x_{gt}\right)}^{2}+{\left(y-y_{gt}\right)}^{2}}{{\left(W_{g}^{2}+H_{g}^{2}\right)}^{*}}){ℒ}_{IoU}$$

where *W*_*g*_ *H*_*g*_ are the size of the smallest enclosing box.

##### 3.2.5.2. WIoU v2

Focal Loss introduces a monotonic focusing mechanism for cross-entropy, which substantially reduces the contribution of easy examples to the loss value. By doing so, it enables the model to concentrate on difficult examples and achieve enhanced classification performance. This is accomplished by constructing the monotonic focusing coefficient $L_{IoU}^{*}$ of $L_{WIoU\;v1}$. However, during the model training process, the gradient gain $L_{IoU}^{*}$ decreases as $L_{IoU}$ decreases. This causes slower convergence in the later stages of training. To mitigate this issue, the introduced $L_{IoU}$ mean is used as a normalization factor, which helps to balance the gradient gain and expedite the training convergence.


(6) $${ℒ}_{WIoU\;v2}={\left(\frac{{ℒ}_{IoU}^{*}}{\bar{{ℒ}_{IoU}}}\right)}^{\gamma }{ℒ}_{WIoU\;v1},\gamma >0$$

Where $\bar{{ℒ}_{IoU}}$ is the running mean with momentum *m*. The normalization factor is dynamically adjusted throughout the training process to ensure that the overall gradient gain remains high. This strategic adjustment effectively addresses the issue of slow convergence during the later stages of training, fostering a more efficient and robust learning environment.

##### 3.2.5.3. WIoU v3

Firstly, an outlier is defined to characterize the quality of the anchor box. A smaller outlier implies a higher quality anchor box. Correspondingly, a smaller gradient gain is allocated to such high-quality anchor boxes, thereby allowing the bounding box regression to concentrate more on anchor boxes of average quality. Allocating smaller gradient gains to anchor boxes with larger outliers effectively mitigates the risk of low-quality examples generating disproportionately large, detrimental gradients. Finally, a non-monotonic focusing coefficient, denoted by β, is constructed and applied to enhance the functionality of WIoU v1.


(7) $${ℒ}_{WIoU\;v3}=\frac{\beta }{\delta \alpha^{\beta -\delta }}{ℒ}_{WIoU\;v1},\beta =\frac{{ℒ}_{IoU}^{*}}{\bar{{ℒ}_{IoU}}}\in [0,+\infty ]$$

### 3.3. Improved network structure YOLOv7-CSAW

**Figure 4:**
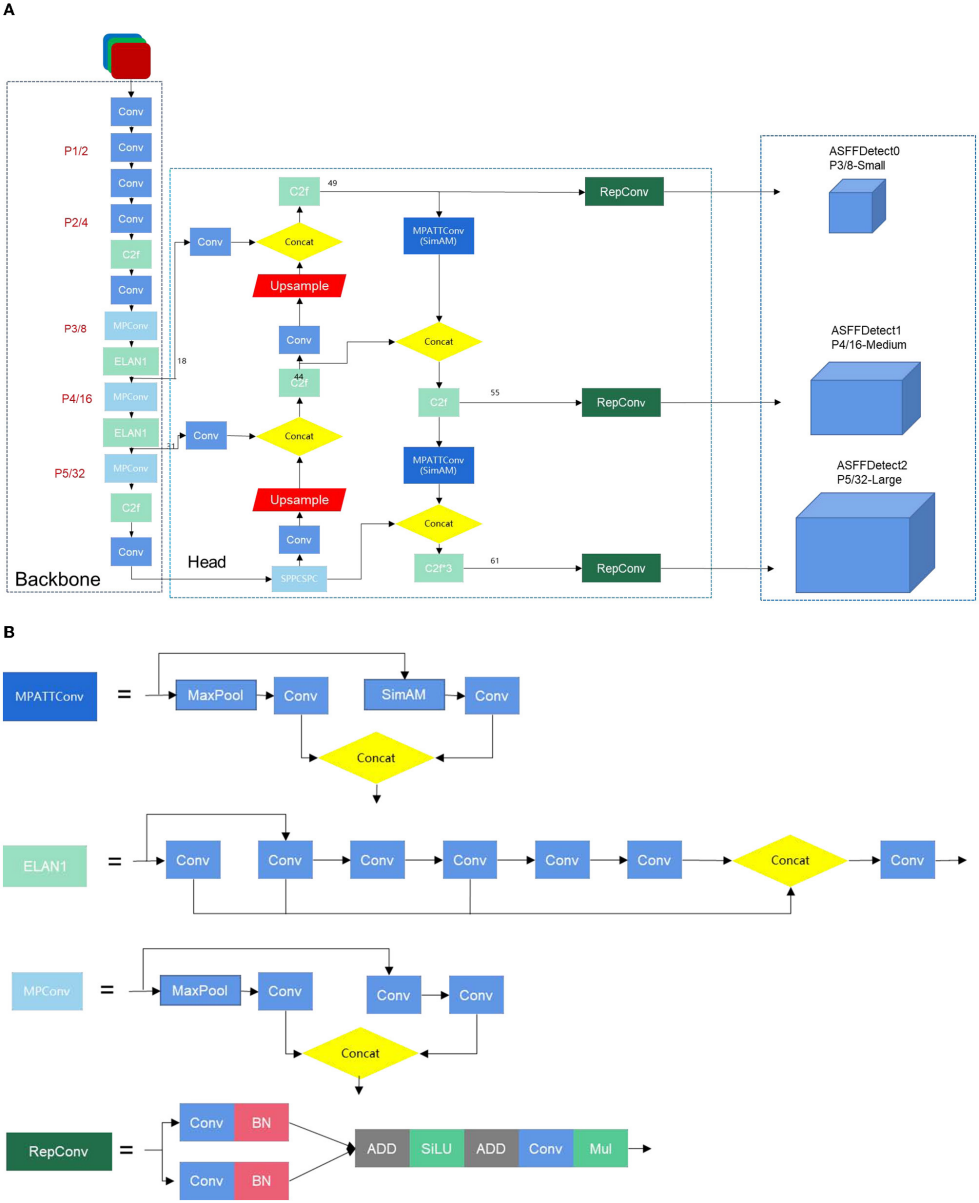
**(A)** The network structure of YOLO-CSAW. **(B)** The network structure of YOLO-CSAW.

Figure 4 presents the network architecture of YOLOv7-CSAW, starting with a description of the main modules. The improved model uses two C2f modules to replace the original ELAN modules in the backbone network. In the head portion, the structure is revamped in the style of RepVGG. In this improved head network, the original four ELAN modules are replaced with C2f, and the SimAM attention mechanism is introduced into MPConv. The original detection head, IDetect, is replaced with ASFF, and the loss function is modified to Wise-IoU v3. In addition, the MPConv module has two branches, both of which serve the purpose of downsampling. The first branch passes through a max-pooling layer, followed by a convolution layer that alters the number of channels. The second branch initially traverses a convolution layer to adjust the channel count, and then proceeds to a convolutional block for further downsampling. The ELAN module is an efficient network structure that controls both the shortest and longest gradient paths, allowing the network to learn more feature representations and exhibit greater robustness. The UPSample module uses nearest-neighbor interpolation for upsampling. The SPP module expands the receptive field, enabling the algorithm to accommodate images of different resolutions by using max-pooling to capture a range of receptive field sizes. The CSP module first divides the features into two parts: one part undergoes traditional processing, and the other part is processed by the SPP structure. Finally, these two parts are merged, effectively halving the computational load, which leads to faster processing speeds and improved accuracy.

In the preprocessing phase, using default anchor boxes might lead to bounding boxes that are too large and cannot accurately capture small objects. By re-generating anchor boxes using the K-means++ clustering algorithm, the size and ratio of the anchor boxes can be optimized according to the actual size and distribution of small objects in the training data. This helps to better adapt to the detection of small objects. By accurately matching anchor boxes with actual objects, the recall and precision of object detection can be improved.

The application of the C2f module in the backbone of the neural network can enhance the ability to perceive context information, improve the expressive power of low-level features, and strengthen multi-scale information. These improvements can enhance the performance of the model's object detection, especially the detection of small objects, which is significantly improved.

Using the SimAM attention mechanism in the head and neck parts of the object detection model can enhance the model's perception of small objects. SimAM can adaptively adjust the weights of the feature map and focus more on the local area of the target. This can improve the positioning accuracy of the target and reduce localization errors.

The ASFF module can fuse features from different levels at the Detect end. Combining semantic information from different levels allows the model to more comprehensively capture the features of the target, thereby improving the performance of object detection. This helps the model better adapt to the characteristics of different targets and changes in scenes, dynamically fuse features at the Detect end, and improve the accuracy and robustness of object detection. At the same time, WIoU considers the size and location information of the target when calculating the loss at the detect end, effectively solving problems such as large localization errors and missed detections.

These five improvement measures each optimize different aspects of small object detection, comprehensively enhancing the model's detection and localization capabilities for small objects.

## 4. Experimental results

### 4.1. Dataset and experimental environment

**Figure 5:**
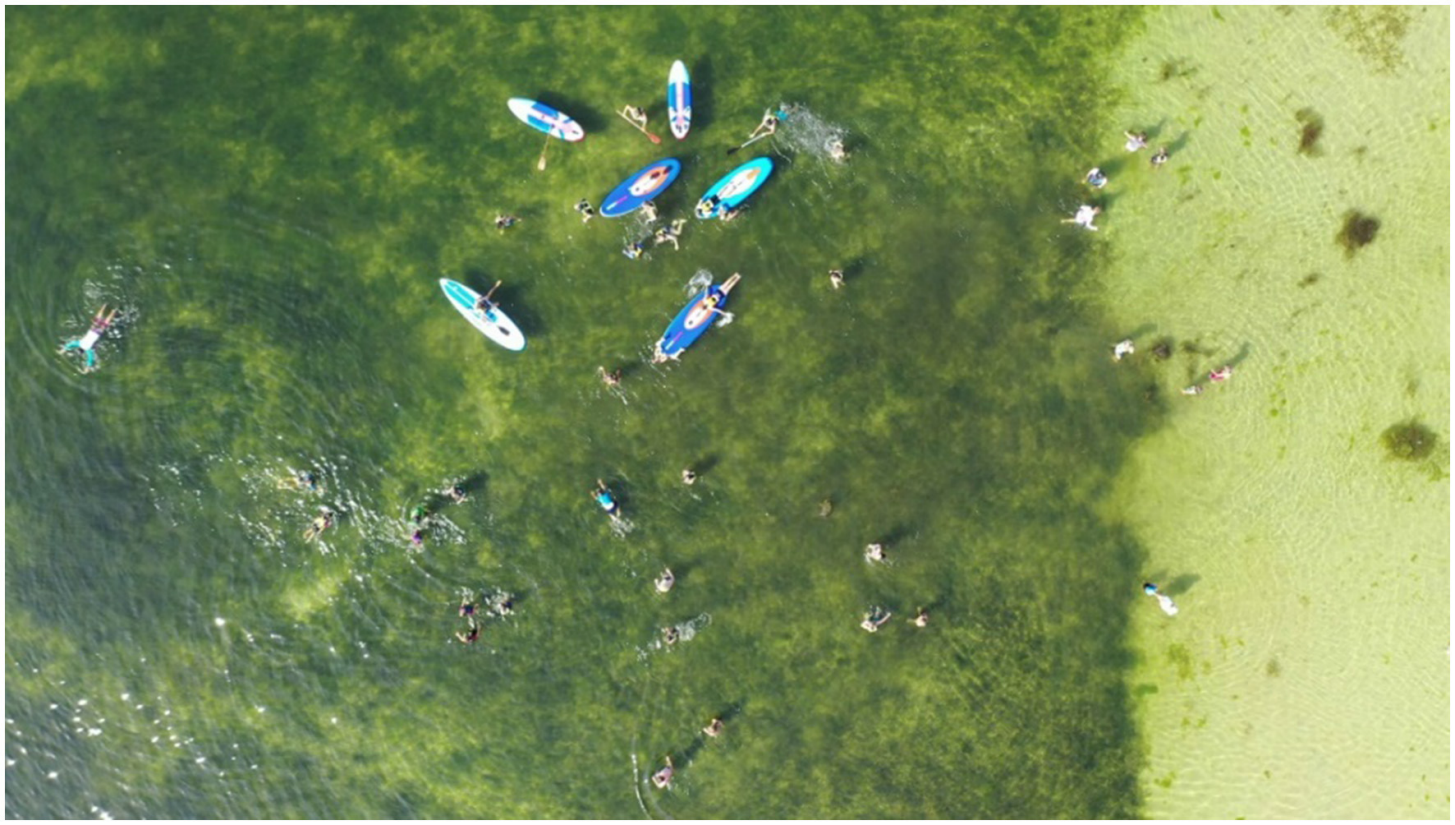
Sample images of the dataset.

The maritime search and rescue dataset (Gasienica-Jozkowy et al., 2021) used in the paper consists of various objects floating on the water surface, captured by different unmanned aerial vehicles with resolutions ranging from 1,280 × 720 to 3,840 × 2,160. It includes 3,647 images and 39,991 target objects, as shown in Figure 5. Over 99% of the object surface areas in the dataset are smaller than 1% of the image area, and there are many crowded images. More than 30% of the images contain 20 instances of objects, and the most crowded image has 50 instances of objects. Table 1 presents the number of object categories in the dataset, which includes six different classes: Human, Board, Boat, Buoy, Sailboat, and Kayak. Furthermore, the objects are classified into large and small categories, with the ratio of large to small targets being ~1:6, and each target category has separate training and validation sets.

**Table 1:** Dataset description.

**Categories**	**Train set**	**Val set**
Human	25,000	8,005
Board	3,083	842
Boat	506	197
Buoy	460	161
Sailboat	115	44
Kayak	904	556
Small object	25,547	8,214
Large object	4,588	1,642

The experimental environment in this paper was Ubuntu 20.04 operating system, NVIDIA A100-SXM4-80GB GPU, 1024GB memory, Intel(R) Xeon(R) Gold 6330 CPU @ 2.00GHz processor, with CUDA 11.4 and Pytorch 1.12.0 software environment.

### 4.2. Model evaluation

In this paper, precision, recall, average precision (AP), mean average precision (mAP), parameters, and floating-point operations (FLOPs) are used as evaluation met-rics for model accuracy. AP represents the area under the precision-recall curve, while mAP represents the mean AP for each category at an IoU threshold of 0.5. The specific formulas are as follows:


$$\begin{array}{l} P=\frac{TP}{TP+FP} \end{array}$$

$$\begin{array}{l} R=\frac{TP}{TP+FN} \end{array}$$

$$\begin{array}{l} AP=\frac{\sum P}{Num\left(objects\right)} \end{array}$$

$$\begin{array}{l} mAP=\frac{\sum AP}{Num\left(class\right)} \end{array}$$

Where TP represents the number of correctly predicted instances of the positive class, FN represents the number of instances of the positive class that were predicted as negative, and FP represents the number of instances of the negative class that were predicted as positive.

### 4.3. Experimental results

All models have an input image size of 1,280 × 1,280, are trained for 300 epochs with the SGD optimizer, a learning rate of 1e-2, momentum of 0.937, and weight decay of 5e-4.

#### 4.3.1. Experimental comparison of different attention mechanisms

**Figure 6:**
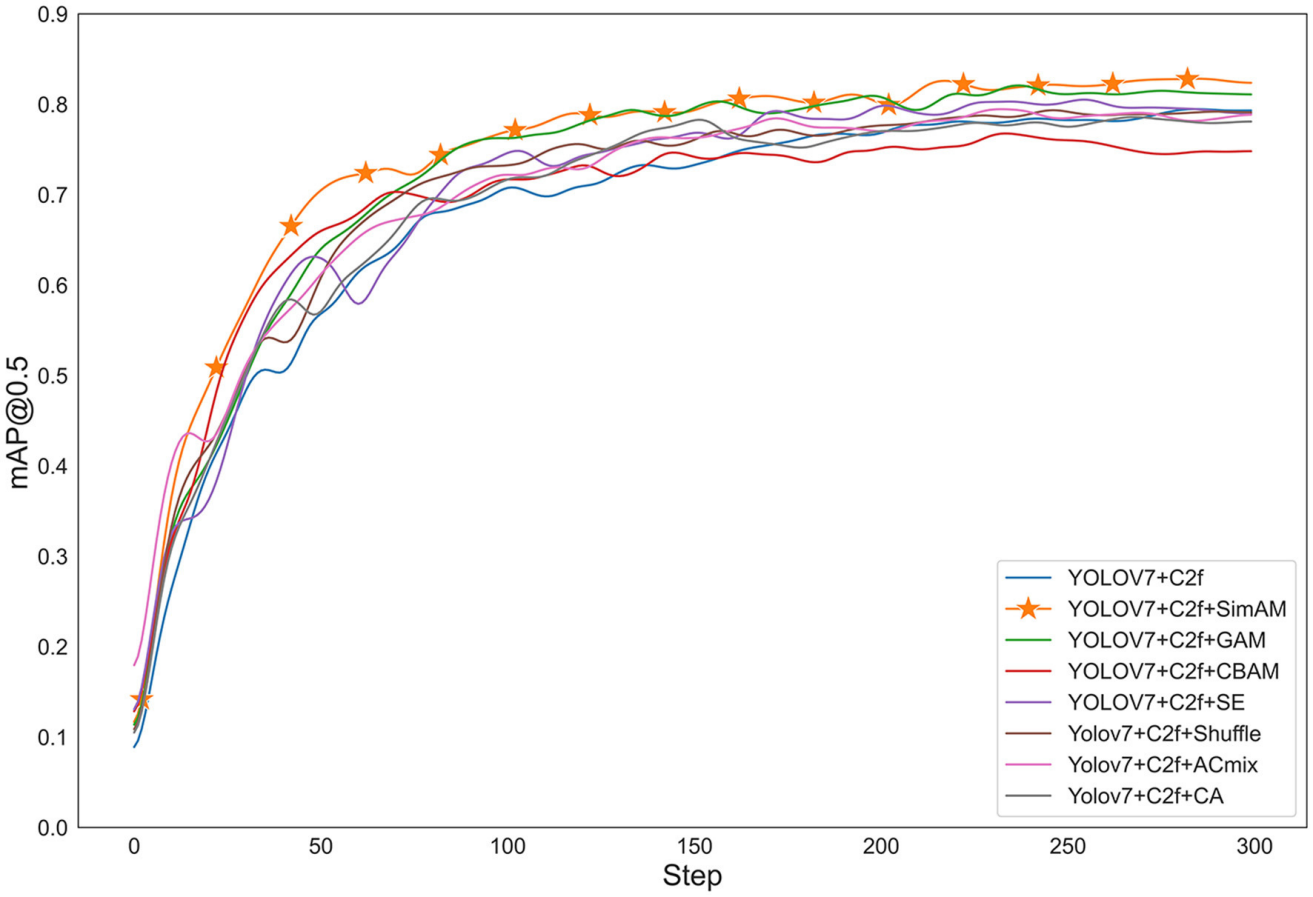
Graph of different attention mechanisms.

To verify the effectiveness of different attention mechanisms, we used YOLOv7+C2f as the baseline model and compared it with various attention mechanisms, including SimAM, CBAM, SE, Acmix (Pan et al., 2022), CA (Hou et al., 2021), GAM (Liu et al., 2021), and Shuffle (Zhang and Yang, 2021). The input image size for all models was 1280 × 1280, and the iteration number was 300. The optimizer used was SGD with a learning rate of 1e-2, momentum of 0.937, and weight decay of 5e-4. From the results shown in Figure 6 and Table 2, SimAM attention mechanism was the most effective, followed by GAM attention mechanism, which improved the accuracy by 5.86% compared to CBAM attention mechanism.

**Table 2:** Detection accuracy of different attention mechanisms.

**Model**	**mAP@0.5**
Yolov7+C2f+SimAM	**83.12**
Yolov7+C2f+CBAM	77.26
Yolov7+C2f+SE	81.28
Yolov7+C2f+Shuffle	79.76
Yolov7+C2f+GAM	82.76
Yolov7+C2f+ACmix	80.30
Yolov7+C2f+CA	80.72

#### 4.3.2. Experimental comparison of different loss functions

**Figure 7:**
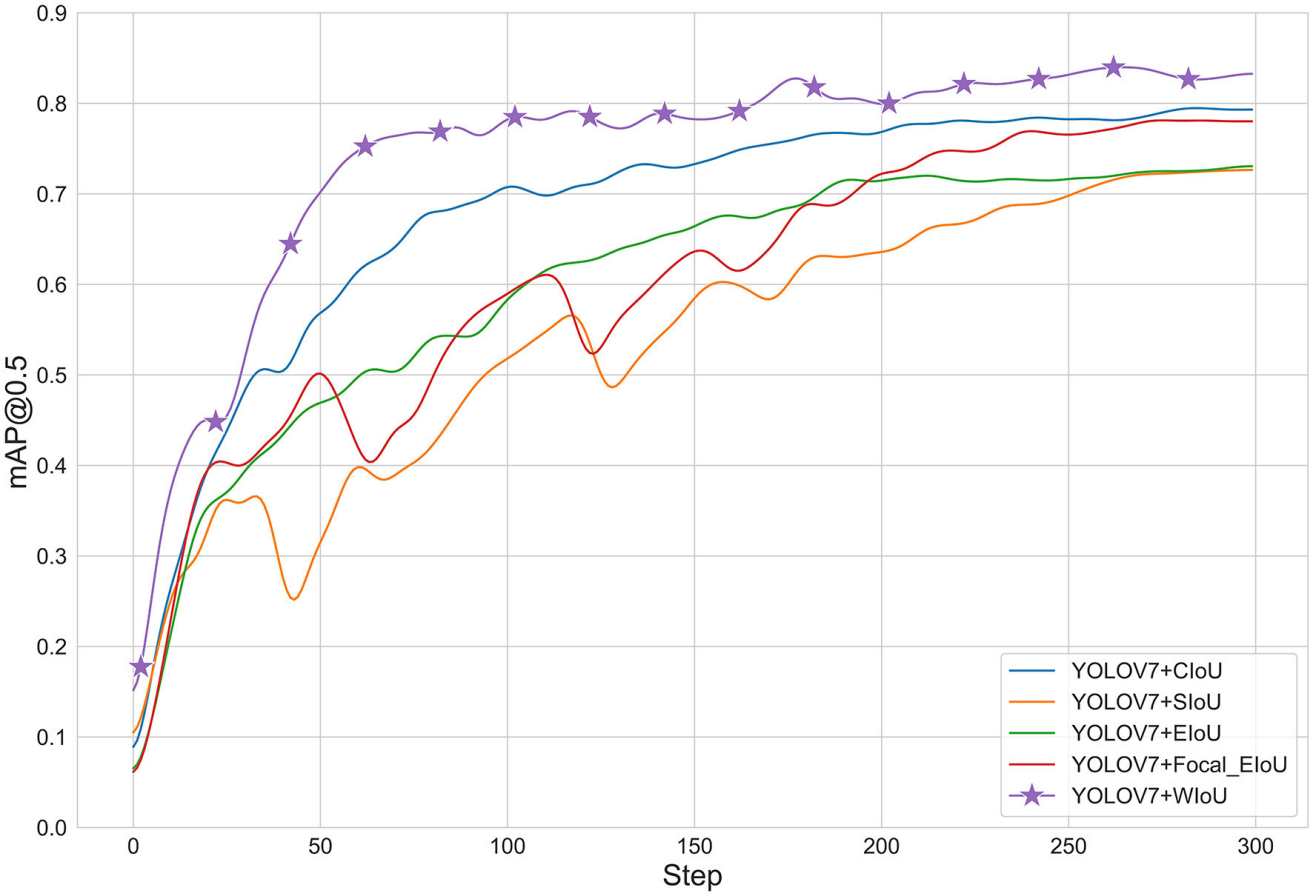
Graph of different loss functions.

To verify the effectiveness of different loss functions, we conducted experiments comparing the CIoU (Zheng et al., 2020), SIoU (Gevorgyan, 2022), EIoU, Focal_EioU (Zhang Y.-F. et al., 2022), and WIoU loss functions. As shown in the Figure 7 and Table 3, it can be seen that the WIoU loss function performs significantly better than the other loss functions, improving the accuracy by 12% com-pared to the SIoU loss function that comes with YOLOv7. The average precision of other loss functions is less than 80%, which also indicates that WIoU can to some extent solve the problem of locating small targets.

**Table 3:** Detection accuracy of different loss functions.

**Model**	**mAP@0.5**
Yolov7+CIoU	75.66
Yolov7+SIoU	72.76
Yolov7+EIoU	73.21
Yolov7+Focal_EIoU	78.39
Yolov7+WIoU	**84.70**

The WIoU loss function plays a pivotal role in improving the performance of the YOLOv7-CSAW model, particularly in terms of accurate localization of small targets in complex marine environments. By addressing large localization errors and minimizing missed detections, the WIoU loss function contributes significantly to the overall effectiveness of the model. To demonstrate the performance improvements achieved through the integration of the WIoU loss function, we compared our YOLOv7-CSAW model with the original YOLOv7 model and other alternative loss functions. The results indicated that the YOLOv7-CSAW model achieved an enhanced mean Average Precision (mAP) of 9.04%, showcasing the effectiveness of the WIoU loss function in reducing localization errors and missed detections. These results highlight the importance of incorporating the WIoU loss function into the YOLOv7-CSAW model, as it significantly improves maritime target detection performance when compared to the original YOLOv7 model and other alternative loss functions.

#### 4.3.3. Ablation experiments

To validate the effectiveness of the proposed improved network, we conducted four ablation experiments, and the results are shown in Table 4. FLOPs indicate the computational complexity of the model, while Params indicate the number of model parameters. The first experiment used YOLOv7 as the baseline; the second experiment added the C2f structure to YOLOv7, improving the detection accuracy by 3.84%; the third experiment added the SimAM attention mechanism based on the second experiment, which further improved the detection accuracy by 3.62%. The fourth experiment combined the adaptive feature fusion and Wise-IoU loss function based on the third experiment, and achieved a 10.73% improvement in detection accuracy compared to the original YOLOv7 algorithm. Table 4 also shows the detection accuracy of Human, Wind, Boat, Bouy, Sailboat, and Kayak. As can be seen from the table, while the original YOLOv7 algorithm achieved an accuracy of more than 0.9 for large targets (Board, Kayak), but the detection accuracy for small targets (Boat, Bouy, Sailboat) was low. YOLOv7-CSAW not only improves the detection accuracy of large targets but also enhances the detection accuracy of small targets. Specifically, the detection accuracy improved by 17.6% for boats and 36.8% for sailboats, resulting in a substantial enhancement.

**Table 4:** Ablation experiments.

**Model**	**Parameter**	**FLOPs(G)**	**Human**	**Board**	**Boat**	**Bouy**	**Sailboat**	**Kayak**	**mAP@0.5**
YOLOv7	35.5M	104.8	0.846	0.982	0.578	0.615	0.570	0.936	75.66
Yolov7+C2f	38.1M	43.7	0.846	0.982	0.637	0.614	0.818	0.863	79.5
Yolov7+C2f+SimAM	38M	43.8	0.890	**0.985**	0.638	**0.643**	0.882	0.899	83.12
YOLOv7-CSAW	58.7M	52.2	**0.896**	0.983	**0.754**	0.614	**0.938**	**0.941**	**86.39**

#### 4.3.4. Comparative experiments with mainstream algorithms

To demonstrate the effectiveness of our proposed method, we conducted experiments on the sea dataset using six classic object detection algorithms, namely YOLOv3 (Redmon and Farhadi, 2018), YOLOv4 (Bochkovskiy et al., 2020), SSD (Liu et al., 2016), Faster-RCNN, RetinaNet (Lin et al., 2017), and Ensemble 3 from reference (Gasienica-Jozkowy et al., 2021). Results are shown in Table 5. Faster-RCNN is a classic two-stage detection method that generates candidate boxes through region proposal networks and then performs object classification and bounding box regression. Other algorithms are one-stage detection methods. From Table 5, it can be seen that the mAP of our proposed method reaches 86.39, which is 7.42% higher than the detection accuracy of the two-stage detection algorithm Faster-RCNN, 6.57% higher than the best one-stage detection algorithm RetinaNet, and 4.23% higher than the ensemble network Ensemble 3. Obtaining these results is reasonable since we added the C2f module in the backbone network to obtain more abundant gradient flow information, used the adaptive feature fusion module in the feature fusion stage to balance the high-level semantic information and spatial position information in the shallow feature map, and provided sufficient semantic information for detecting small targets. Additionally, we added the SimAM attention mechanism in the Head part to enhance the extraction ability of shallow, intermediate, and high-level features.

**Table 5:** Comparative experiments with mainstream algorithms.

**Detector**	**Backend**	**AP@0.5**
YOLOv3 (Redmon and Farhadi, 2018)	Darknet53	68.58
YOLOv4 (Bochkovskiy et al., 2020),	CSPDarknet53-PANet-SPP	71.13
SSD300 (Liu et al., 2016)	MobileNet v2	41.40
Faster-RCNN (Yabin et al., 2020)	ResNet101 + FPN	78.97
RetinaNet (Lin et al., 2017)	ResNet50	79.82
Ensemble 3 (Gasienica-Jozkowy et al., 2021)	F-RCNN (ResNet101) RetinaNet (ResNet50)	82.16
YOLOv7 (Wang et al., 2023)	CBS+E-ELAN+MP	75.66
YOLOv7-CSAW	CBS+E-ELAN+MP	86.39

In addition, we also conducted comparative tests for two major categories, small and large objects, as shown in Table 6. From the table, it can be seen that the overall performance of YOLOv7-CSAW is better than that of the original YOLOv7 model, especially with a 4.2% increase in accuracy in the small object category, which indicates that the proposed algorithm has certain detection capabilities for small objects.

**Table 6:** Two-category comparative experiment.

**Detector**	**AP@50**		
	**Small object**	**Large object**	**All**
YOLOv7 (Wang et al., 2023)	0.854	0.914	0.886
YOLOv7-CSAW	0.896	0.918	0.907

#### 4.3.5. Algorithm analysis

To compare the feature extraction abilities of the proposed method in small object detection, we used the Grad-CAM (Gradient-weighted Class Activation Mapping) method to display the heatmaps of detected objects. The Grad-CAM algorithm can use class gradients to help analyze the network's attention regions for a particular class, and visualizing the network's attention regions can provide important insights into whether the network has learned the correct features or information for image classification.

**Figure 8:**
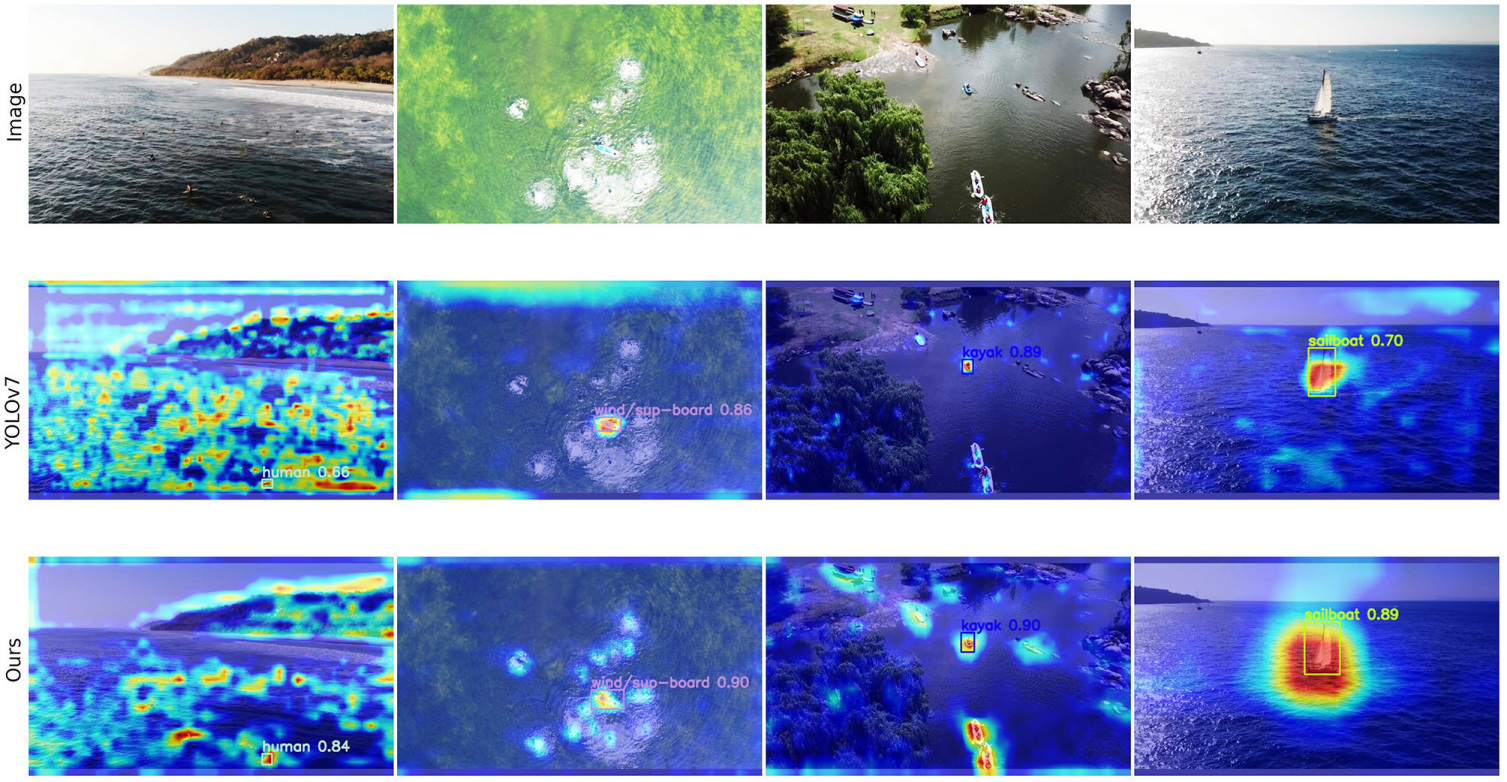
Grad-CAM diagram of YOLOv7 and YOLOv7-CSAW.

Figure 8 shows the Grad-CAM images for YOLOv7 and YOLOv7-CSAW. The brighter areas in the figure indicate the regions that the network paid more attention to. To provide a comprehensive analysis of the comparison, we selected images of different categories and different sizes of detected objects, all of which were correctly identified by both networks. The Human and Board belong to small objects, while Kayak and Sailboat belong to large objects. In the YOLOv7-CSAW method, the detection results for human, board, kayak, and sailboat targets show improvements of 0.18, 0.04, 0.01, and 0.11, respectively, compared to the baseline model. The results of small object detection show that the YOLOv7-CSAW model can extract more features for small objects, as reflected by the heat map covering more parts of the small objects and being brighter and more concentrated. The YOLOv7-CSAW model is less affected by interference in areas without objects and can increase its attention to adjacent or similar objects through attention mechanisms and feature fusion. Large object detection is relatively easy and has a high accuracy rate. Comparing the results of large object detection, we found that the YOLOv7-CSAW model pays more extensive attention to possible regions and can give greater and more focused attention to other similar objects during the detection of Kayak and Sailboat. Therefore, YOLOv7-CSAW has better feature extraction and anti-interference abilities for small objects and a more extensive attention range during the detection process.

**Figure 9:**
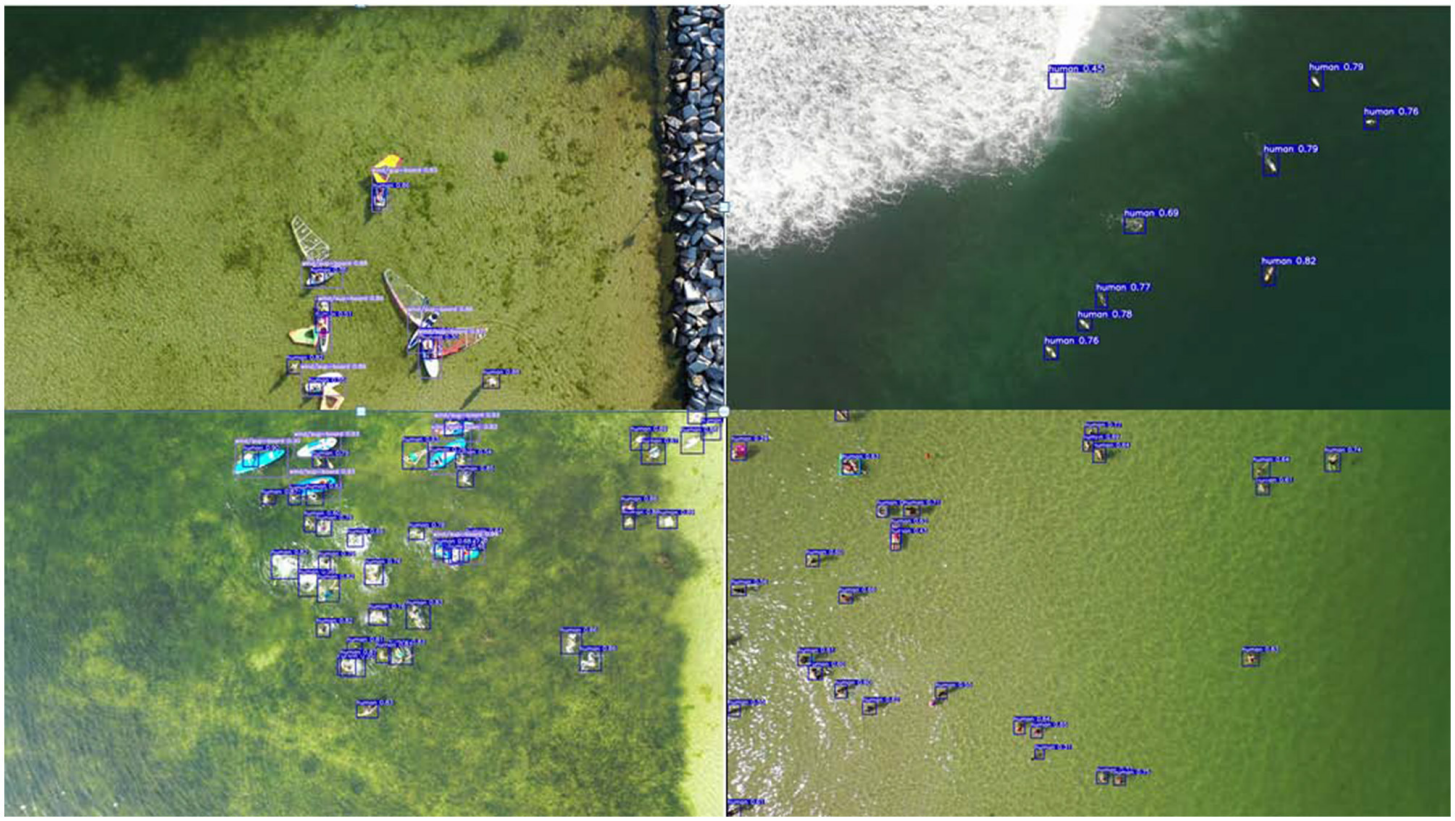
Detection results with the YOLOv7-CSAW algorithm.

Figure 9 illustrates the impressive detection capabilities of the YOLOv7-CSAW al-gorithm across a range of diverse environmental conditions. The following descriptions highlight its capabilities in different settings:

- Calm sea conditions: In situations with minimal wave activity and clear visibility, YOLOv7-CSAW accurately identifies and locates small marine targets, such as boats, swimmers, and floating debris.
- Rough sea conditions: Even in the presence of significant wave action and turbulent waters, the algorithm effectively distinguishes between objects of interest and the surrounding water, maintaining high accuracy in target detection.
- Low-light conditions: YOLOv7-CSAW is capable of detecting objects under low light or at dusk, thanks to its robust feature extraction and attention mechanisms, which help to identify targets even in challenging lighting situations.
- High-contrast environments: The YOLOv7-CSAW algorithm manages to effectively detect targets in settings with significant contrast, such as bright sunlight or sharp shadows, by leveraging its adaptive feature fusion network to emphasize relevant features while suppressing background noise.

Overall, the YOLOv7-CSAW algorithm showcases its strong performance and adaptability in various environmental conditions, making it a valuable tool for marine target detection in diverse scenarios. However, there are circumstances in which our algorithm fails to perform successful detection, primarily when dealing with small objects, distant objects, or in situations with poor lighting conditions. The main reason for these detection failures is the use of a training dataset obtained from a drone's vertical field of view. When employing surveillance cameras set at various angles, like those monitoring lakeshores or providing security for seaside hotels, the model may occasionally miss a few detections. Furthermore, in oceanic environments, there exist features such as rocks and reefs that can mimic the shapes of Boats, Buoys, or Sailboats. This resemblance can potentially cause false detections.

## 5. Conclusion

In order to address the issue of missed and false detections of small targets in complex marine environments, this paper proposes an improved model, YOLOv7-CSAW, based on the original YOLOv7. The YOLOv7-CSAW model enhances the backbone network with a C2f module, which allows for richer gradient flow information while maintaining a lightweight structure. Additionally, the inclusion of the parameter-free attention mechanism, SimAM, bolsters the model's ability to perceive small target features. The improved feature fusion network, termed ASFF, compensates for the lack of high-level semantic features in small targets. Furthermore, the WIoU loss function effectively resolves issues related to large localization errors and missed detections, ultimately improving the model's generalization ability. Through a series of tests such as attention mechanism evaluation, loss function analysis, ablation experiments, and comparative experiments with mainstream algorithms using a marine dataset, the results demonstrate that the proposed YOLOv7-CSAW model exhibits superior detection performance on small targets, along with enhanced generalization ability and robustness compared to the original YOLOv7. In future research, we plan to explore model compression and lightweight design, with the aim of deploying the model on mobile and embedded devices while balancing accuracy and speed.

## Data availability statement

The original contributions presented in the study are included in the article/supplementary material, further inquiries can be directed to the corresponding author.

## Author contributions

QZ: conceptualization, resources, and writing—original draft preparation. ZW: methodology, data curation, and writing—review and editing. KM: software and validation. PS: formal analysis and investigation. All authors have read and agreed to the published version of the manuscript.

## Footnotes

^1^China Maritime Search and Rescue Center. Available online: https://zizhan.mot.gov.cn/sj2019/soujiuzx.

^2^ https://github.com/ultralytics/ultralytics
